# Evaluating circulating tumour cell enrichment techniques to establish an appropriate method for clinical application in glioblastomas

**DOI:** 10.3389/fneur.2024.1358531

**Published:** 2024-02-28

**Authors:** Hannah R. Barber, Claire M. Perks, Kathreena M. Kurian

**Affiliations:** ^1^Brain Tumor Research Centre, Bristol Medical School, Translational Health Sciences, Southmead Hospital, University of Bristol, Bristol, United Kingdom; ^2^Cancer Endocrinology Group, Bristol Medical School, Translational Health Sciences, Southmead Hospital, University of Bristol, Bristol, United Kingdom

**Keywords:** liquid biopsy, brain tumour, circulating tumour cells, enrichment methods, glioblastoma, blood test

## Abstract

Brain tumours reduce life expectancy for an average of 20 years per patient, the highest of any cancer. A third of brain tumour patients visit their GP at least five times before diagnosis and many of those are diagnosed late through emergency departments. A possible solution to this challenge is to utilise a “liquid biopsy” blood test designed for circulating tumour cells (CTCs). Such a test could be applied at a primary healthcare centre, contributing to informed decision making for diagnostic imaging referrals. Furthermore, it could also be applied at secondary health care centres for the ongoing monitoring of disease recurrence. There is increased interest in CTC enrichment methods as a potential approach for faster diagnosis and monitoring of disease progression. The aim of this review to compare four CTC enrichment methods - OncoQuick^®^, Screen Cell^®^, pluriBead^®^ and Cell Search^®^ – with the objective of identifying a suitable method for application in the clinical setting for the isolation of CTCs from glioblastomas.

## 1 Introduction

Globally, it was estimated that 308,102 people were diagnosed with a primary central nervous system (CNS) tumour in 2020, with incidence rates projected to rise by 6% between 2014 and 2035 (1, 2). Brain tumours cause more fatalities in children and adults under the age of 40 than any other cancer, reducing the life expectancy by an average of 20 years per patient, the highest of any cancer (3, 4).

Despite advances in surgical resection, chemotherapy and radiotherapy, around only 13.5% of adults survive brain tumours for five or more years after diagnosis (5). Data compiled by the Brain Tumour Charity found that a third of brain tumour patients had visited their GP at least five times before diagnosis (6, 7). Furthermore, over 50% are diagnosed via emergency departments rather than the GP; many of those patients presenting later in the course of the disease with large, inoperable tumours (8, 9).

Presently diagnosis and disease monitoring rely on access to imaging in secondary care, which is costly and overburdened: with 230,000 patients waiting more than a month for test results (10). Imaging can also intermittently produce false positive results due to non-malignant inflammatory changes mimicking tumour recurrence (11). Subsequent to imaging, the patient will undergo neurosurgery, during which a diagnostic tissue biopsy is taken. However, this biopsy provides static information that becomes obsolete as the cancer evolves. Different sub-clones expressing altered targetable biomarkers may emerge within the cancer during the course of the disease, highlighting the limitations of relying solely on static biopsy data (12). A better understanding of intertumoral heterogeneity is required to inform mechanisms of tumour resistance to therapies (13, 14). Consequently, there is an urgent need to utilise innovative methodology to improve patient diagnosis and overall survival.

A potential solution is to utilise a liquid biopsy assay for circulating tumour cells (CTCs) in peripheral blood samples from brain tumour patients. Not only are these simple blood tests low in cost and minimally invasive; they could be implemented in both the primary or secondary care setting (15). This has the potential to expedite diagnosis, monitor tumour genomic changes through serial samples and detect early relapse or resistance to current therapies (16, 17).

The benefits of CTC detection have been widely explored in other malignancies such as breast, colorectal, prostate, gastric, bladder, melanoma and small and non-small cell lung carcinoma cancer (18–26). In 2013, the LANSCAPE trial investigated CTC levels in breast cancer patients, with metastases to the brain, before and after treatment with lapatinib and capecitabine at 21 days, in Her2 positive tumours (27). The trial demonstrated a correlation between CNS metastasis response, outcome, and early CTC clearance under targeted treatment of Her2 positive, metastatic breast cancer (27). CTC count has also been shown to predict progression-free survival and overall survival in non-small cell lung carcinomas after multivariate analysis (23).

Using MTW9 carcinomas, (28) demonstrated that the presence of large numbers of tumour cells in the blood is not, by itself, a sufficient condition for metastatsis to occur. Multiple studies have similarly demonstrated that despite the detection of a high number of cancer cells in the blood, as few as 0.01% of CTCs develop into secondary tumours (29–31). CTC intravasation can occur through active and passive shedding (32). Bockhorn et al. (33), identified the loss of CD44 and α3 integrin in CTCs shed from renal cell carcinoma. Both CD44 and α3 integrin play a role in cell adhesion and a reduction makes it much easier for the cells to pass into the blood stream (34, 35). Blood vessels created by angiogenesis are immature, malformed and leaky with detached endothelial cells and an irregular or missing basement membrane (36, 37). Although it is not fully understood how these abnormalities affect intravasation it most likely helps with this process and could also account for non-viable cells, as well as viable cells, being leaked into the blood stream (38, 39). Proliferating cells have also been shown to compress and collapse intra-tumour blood vessels, which would enable the tumour cells to passively enter the blood stream (38, 39).

Throughout tumour progression there is active cross-talk between the tumour cells and micro-environment (30). This signalling is mediated by cell-to-cell interactions and cytokine/growth factors. Morphological changes which support metastasis are triggered by this signalling (40). Neurons, a crucial component of the glioma microenvironment, have been shown to regulate malignant growth in an activity dependent manner (41, 42). Synaptic communication is suggested to occur through AMPA (α-amino-3-hydroxy-5-methyl-4-isoxazole propionic acid) receptors, particularly the glutamate receptor (43). Glutamate, a key neurotransmitter, is considered a potential growth factor for glioma development (44).

Many studies have supported the hypothesis that neoplasms are heterogeneous and there is a distinct sub-population of tumour cells, with differing angiogenic, invasive and metastatic properties (45). This distinct subgroup can use epithelial-mesenchymal transition (EMT) to become increasingly motile, which in turn enables them to migrate to the vascular system through growth factor and nutrient gradients (46, 47). In contrast to the passive model described above these cells have been shown to actively migrate, passing either paracellularly through the endothelial cell junction or transcellularly through the endothelial cell body into the blood stream (48). These highly metastatic cells have been shown to produce matrix metalloproteinases, which actively digest the interstitial matrix and basement membrane, enabling them to pass through the tissue into the blood stream (49).

CTC arrest can be triggered by several obstacles within the bloodstream, including entrapment by capillaries; reduced diameter dimensions and biomechanical constriction forces of the capillary lumen, which have been shown to severely deform the cell cytoplasm and nucleus thus triggering cell death (50, 51). It has been suggested that capillary constriction can reduce the potential for CTCs to enter the vessels by as much as 90% (52). The role of capillary entrapment is less clear because entrapment may also be important for metastatic progression, enabling CTCs to adapt to the new environment, facilitating invasion and colonisation at the metastatic site (53, 54). In order to survive in the bloodstream CTCs must also evade hemodynamic shear forces and the immune system (55, 56). However, increasing evidence is emerging that CTCs are not as mechanically fragile as first thought and in fact can withstand fluid shear stresses encountered through circulation (57). CTCs have been shown to induce platelet activation and aggregation to protect their survival in the blood stream. Mounting evidence has also validated this interaction as a key feature of metastasis (58–61).

Originating from glial cells it is estimated that gliomas account for 75% of all primary malignant brain tumours (62). Glioblastomas (GBMs), the most aggressive and common glioma, are associated with dismal prognosis and rapid recurrence, despite multimodal therapies (62, 63). GBM cells are highly migratory and extensive infiltration of these cells into the brain parenchyma makes remedial surgical resection almost impossible (64). Systemic metastases from GBMs however are incredibly rare, 0.5% metastasise compared to 10–45% of other primary cancers that metastasise to the brain. It is thought that the brain’s distinct microenvironment, containing the blood brain barrier and stem cell niches, significantly influences this rate (65).

The permeability of the blood–brain barrier is associated with GBM progression, heightened intravasation chances, and is suggested to be due to the disruption of endothelial/ astrocytic interaction and impaired vessel formation (66). Davis (67), reported the first ever case of GBM metastasis. Since then this number has increased progressively: this is thought to be due to improvements in imaging and patient survival (68). Metastatic GBM cells can spread through blood and lymphatic vessels (69). Onda et al. (70), undertook autopsies on 51 patients who had died from GBM and found that 14 of the 51 cases had dissemination by cerebral fluid.

Since 2014, there has been substantial progression in CTC isolation and characterisation from high grade glioma patients (Table 1). Sullivan et al. (71), found isolated CTCs had elevated markers, which are associated with the more aggressive mesenchymal subtype. GBMs can be divided into 4 subtypes: proneural, neural, classical and mesenchymal (82). The mesenchymal subtype, characterized by higher migratory capabilities, is associated with worse prognosis and is strongly linked to GBM metastases and recurrences (83, 84). Microglia have been shown to induce mesenchymal status through the tumour necrosis factor alpha (TNF-α)/nuclear factor kappa-light-chain-enhancer of activated B cells (NF-κB) pathway. Additionally, hypoxia has been shown to induce transition and increase stem cell markers in GBM cells (84, 85). Multiple subtypes coexist within the same tumour, and mesenchymal transition is thought to occur late in GBMs, resulting in a more aggressive, invasive and recurrent tumour (86, 87).

**Table 1:** Summary of publications and the methods used to isolate and characterize circulating tumour cells (CTCs) in high grade glioma patients.

Publication	CTC isolation method	CTC characterisation	Results *n* = number of patients with CTCs	Limitations
Publications positively identifying CTCs in glioma patients				
(71)	Enriched from GBM patients. Blood processed through a CTC-iChip® (magnetically tagged CD45 and CD16). Immunofluorescence guided single cell micromanipulation used to isolate CTCs (EGFR, c-MET and CDH11).	IHC glioma marker panel (SOX2, Tubulin, beta-3, EGFR, A2B5, and c-MET). FISH used to determine EGFR gene amplification in CTCs from known amplified cases.	*n* = 28/87 RNA-ISH demonstrated an enrichment for mesenchymal transcripts and a reduction of neural differentiation markers.	Relies on immunostaining for CTC characterisation, may be missed due to CTC heterogeneity. Could not determine whether surgical or radiation induced disruption of BBB enhances CTC dissemination.
(72)	Enriched from high grade glioma patients. Blood samples centrifuged in OncoQuick® tubes.	Incubated with telomerase-responsive adenoviral probe (via GFP expression). Secondary IF (Nestin and EGFR).	*n* = 8/11 pre-radiotherapy *n* = 1/8 post- radiotherapy EGFR amplification in CTCs correlates with solid tumours.	Limited pilot data. Need more serial measurements throughout the treatment and disease for each patient. Telomerase is elevated in other tumour histologies.
(73)	Enriched from GBM patients. MNC isolated by ficoll density gradient centrifugation. Cytospins prepared from MNC. GFAP positive single cells isolated by micromanipulation.	Chromogenic and fluorescent IHC (GFAP, CD45 and EGFR). Further characterisation of CTC and associated tumour: comparative genomic hybridization, sequence analysis and FISH.	*n* = 29/141 Observed association between EGFR amplification and release of CTCs. Common genomic abnormalities in CTCs and GBM tumours.	Low detection rates. GFAP can be detected in other cell types.
(74)	Enriched from glioma patients (7 subtypes). Subtraction enrichment for removal of white and red blood cells.	Interphase FISH for detection of chromosome 8 polyploidy.	*n* = 24/31 CTCs could be detected in 7 subtypes of glioma. No difference between low and high grade. CTCs could be used to distinguish tumour from necrosis. CTCs could be used to predict tumour recurrence.	Limited pilot data.
(75)	Enriched from patients with focal intracranial lesions – GBM and Diffuse Large B-Cell Lymphoma. Density gradient centrifugation followed by short-time culture on chamber-slides.	IF for Vimentin. Fixed with Cytofix aerosol preparation, stained with Hematoxylin and Eosin for pathological analysis.	*n* = 2/2 No obvious pathological difference between excisional and liquid biopsy in diagnostic evaluation of space-occupying brain lesions. Early detection of tumour recurrence.	Limited dataset.
(76)	Enriched from glioma patients (grade II-IV). Transduced with HSV1-hTERT-GFP.	Flow cytometry (CD45-/GFP+) >3 CTCs per 4 mL blood	>3 CTCs per 4 ml blood *n* = 11/23 grade II *n* = 9/13 grade III *n* = 12/15 grade IV The positive rate of CTCs in gliomas rose progressively with advancing stage of disease.	Small sample size. Enrichment and characterisation process is lengthy.
(77)	Enriched from recurrent or progressive GBM patients. Parsortix microfluidic cassette technology (label free physical capture) to detect single and clustered CTCs in an antigen independent manner.	Captured cells stained with a cocktail of antibodies (EGFR, Ki67, EB1 and CD45 to exclude WBCs).	*n* = 7/13 First evidence that circulating GBM can overcome the blood brain barrier and reach peripheral circulation.	Limited pilot data. Relies on immunostaining for CTC characterisation, may be missed due to CTC heterogeneity.
(78)	Enriched from glioma patients (grade II-IV). Magnetic beads coated with VAR2CSA malarial protein (rVAR2) to capture CTCs through the protein oncofetal chrondroitin sulfate.	Fluorophore-conjugated rVAR2, CD45 and CD66b was used for microscopic detection of the captured cells. CTCs classified as rVAR2+/CD45-/CD66b- Targeted whole exome sequencing identified gene with cancer indicative mutations (RB1, TP53/EPM2AIP1, and TP53/ALK).	10/10 No correlation between the number of CTCs and WHO grade.	Limited dataset.
(79)	Enriched from glioblastoma patients. Spiral microfluidic technology.	Characterisation with immunofluorescence for GFAP and cell surface vimentin. CD45 was used to differentiate white blood cells. DNA FISH was used for the detection of EGFR amplification.	13/20 patients (9/20 before surgery and 11/19 after surgery). Patients with CTC counts equal to 0 after surgery had significantly longer recurrence free survival.	Limited cohort. Lack of specific markers for the characterisation of CTCs.
(80)	Enriched from GBM, astrocytoma and low grade glioma patients. Sized based separation protocol and MetaCell® tubes.	Vital fluorescent staining microscopy with defined characteristics (nuclear size and contour, visible cytoplasm, prominent nucleoli, high nuclear-cytoplasmic ratio, fatty cytoplasm, and mitochondrial network presence) (*n* = 18). Next,-generation sequencing (*n* = 8).	*n* = 18/18 CTCs successfully cultured. More mutations detected in CTC samples compared with paired primary tumour. Highlights potential for CTCs to be used for glioma diagnosis, patient monitoring and recurrence.	Limited dataset.
(81)	Primary diffused glioma patients. Biocompatible parylene polymer membrane with a pore diameter of 8 μm under a high flow rate, enriching CTCs without requiring tumour cell-specific capture.	Characterisation using antibody cocktail, SOX2, Tubulin, beta-3, EGFR, A2B5 and c-MET, with a series of criteria of malignant features.	Recurred glioma = 7/8 36/42 had detectable CTC. CTCs higher in astrocytomas compared to oligodendrogliomas. Large number of CTC-WBC clusters detected and could help monitor. Recurrence. No difference noted in glioma subtype. CTC level related to P53 mutation, IDH1 status and poor outcome. Resection may promote CTCs in gliomas.	Limited cohort.

GBM, glioblastoma; EGFR, epidermal growth factor receptor; c-Met, mesenchymal-epithelial transition factor; CDH11, Cadherin 11; IHC, immunohistochemistry; SOX2, sex determining region Y-box transcription factor 2; FISH, fluorescence *in situ* hybridization; RNA-ISH, ribonucleic acid *in situ* hybridization; BBB, blood brain barrier; GFP, green fluorescent protein; IF, immunofluorescence; MNC, mononuclear cells; GFAP, glial fibrillary acidic protein; HSV1, Herpes Simplex Virus Type 1; hTERT, human telomerase reverse transcriptase; EB1, end binding protein 1; WBCs, white blood cells; RB1, Retinoblastoma 1; ALK, anaplastic lymphoma kinase; WHO, World Health Organisation; IDH1, isocitrate dehydrogenase 1.

A study by (72), determined that genomic abnormalities not only correlate between isolated CTCs and the tumour of origin, but also revealed the maintenance of epidermal growth factor receptor (EGFR) amplification in CTCs, indicating sustained growth potential. EGFR also promotes stemness in GBM cells (88). Although cohort numbers were small, results suggest that CTC detection could be used to identify GBM patients with a large tumour or those at risk of recurrence (72). In another study, utilising sensitive immunocytochemical detection, with glial fibrillary acidic protein (GFAP) as a marker for CTCs in peripheral blood cytospin preparations, putative CTC cells were detected in 29 out 141 GBM patients (73). Furthermore, these reputed CTCs were more frequently detected in patients with EGFR gene amplification in the corresponding tumour tissues (73).

In 2016 (74), detected 7 different glioma subtypes in peripheral blood samples using an integrated cellular and molecular approach. Clinical data revealed that CTC detection was superior to MRI in monitoring treatment response and differentiating radionecrosis (74). This study identified nonhematogenic aneuploid circulating aneupolid cells in seven diverse subtypes of brain glioma and reported their significance. This has been further supported by Li et al. (89), who detected and characterised aneuploud circulating rare cells in glioma patients and demonstrated their unique clinical significance.

Malara et al. (75), captured CTCs in a 67-year-old GBM patient pre- and 2 months post-surgery. Interestingly, the post-surgery sample showed a higher number of CTCs. Unfortunately, the patient experienced tumour recurrence 9 months after the sample, and succumbed to the disease 5 months later (75).

Zhang et al. (76), found that the positive rate of CTCs in gliomas increased progressively with the advancing stage of glioma. They utilised cell surface marker independent technology based on telomerase specific, replication-selective oncolytic herpes-simplex-virus-1, which identifies viable CTCs from a wide range of malignancies. The first evidence of CTC clusters was confirmed by (77), who noted them in 53.8% of progressive GBM patients. Bang-Christensen et al. (78), successfully isolated CTCs in every blood sample processed with magnetic beads coated with VAR2CSA malarial protein (rVAR2), which detected CTCs through the protein oncofetal chondroitin sulfate. Spiral microfluidic technology was used by (79), to successfully isolate CTCs from GBM patients. The study also demonstrated that patients with CTC counts equal to 0 after surgery had significantly longer recurrence free survival.

A sized based separation protocol with MetaCell® tubes was used by (80), to detect more mutations in CTC samples compared with the paired primary tumour. Qi et al. (81), used biocompatible parylene polymer membranes, with a pore diameter of 8 μm under a high flow rate, to enrich CTCs without requiring tumour cell-specific capture. Qi et al. (81), found CTC numbers to be higher in astrocytoma samples compared to oligodendroglioma samples. A number of CTC-white blood cell clusters, which could be used to monitor recurrence, were also detected in the study. Qi et al. (81), noted no difference in glioma subtype but in contrast found that resection could promote CTCs. It was also found that the level of CTCs was related to p53 mutation, isocitrate dehydrogenase 1 (IDH1) status and poor outcome (81).

CTC isolation techniques can be divided into 2 broad groups: physical and biological (90). Physical properties include separation by size, elasticity and surface charge (91). Methods used include density gradient centrifugation, microfiltration, microfluidics and dielectrophoresis (92). Antibodies with conjugated magnetic or non-magnetic beads are used to separate the CTCs through their biological properties. This can be either through positive selection, CTCs targeted directly, or negative selection, blood cells for example, are targeted and removed through this method (93).

The aim of this review is to assess and compare four commercially available methods for CTC enrichment: OncoQuick®, Screen Cell®, pluriBead® and Cell Search®. By analysing performance metrics and clinical adaptability, the objective of this study is to provide guidance in selecting a suitable method for potential translational application in the clinical setting, with a primary focus on the isolation of CTCs from GBMs. Commercially available methods were intentionally selected in this study to facilitate easier implementation in the clinical setting.

Each method applies a distinct enrichment technique. The Cell Search® system uses anti-epithelial cell adhesion molecule (EpCAM) conjugated with magnetic beads to isolate CTCs. This system is among the most widely used CTC enrichment techniques, as it is the only CTC detection system approved by the Food and Drug Administration in the United States for the enumeration of CTCs in metastatic colorectal, prostate, and breast cancers (94). Pierga et al. (27), used the Cell Search system to demonstrate that CTCs can be used as early predictive markers for poor overall survival and progression free survival in metastatic breast cancer patients. The study also demonstrated the use of CTCs in monitoring treatment benefit. The downside to this system is that it solely relies on EpCAM for detection. Therefore alternative methods have been developed.

The OncoQuick® method, which uses density gradient centrifugation has been shown to yield higher relative tumour enrichment when compared to standard to the standard density gradient centrifugation system Ficoll (95). In addition to CTC detection in gliomas, the OncoQuick® method has successfully isolated CTCs in studies involving colorectal cancer, melanoma and breast cancer patients (18, 96, 97). The isolation of CTCs through pluriBead® involves the use of non-magnetic beads coupled with monoclonal antibodies specific to the CTC surface antigens. Pierzchalski et al. (98), successfully validated this system for simultaneous separation of CD4+ and CD8+ cells from human EDTA-blood samples. The ScreenCell® method, which captures CTCs through size isolation, determined CTCs in patients with a less favourable stage III laryngeal squamous cell carcinoma (99). The ScreenCell® method has also isolated CTCs from urinary bladder, metastatic prostate and colorectal cancer (100–102).

## 2 Methods (including materials and equipment)

### 2.1 Cell culture

To evaluate the four enrichment techniques OncoQuick®, Screen Cell®, Cell Search® and pluriBead®, the human GBM cell line U251 MG, obtained from Sigma Aldrich (Irvine, North Ayrshire, UK), was spiked at various densities in healthy donor ethylenediaminetetraacetic acid (EDTA)-anticoagulated whole blood samples. Normal whole blood was collected using the standard venepuncture technique.

Table 2 provides information on the human cell lines used in this review, including the corresponding culture media used, and the enrichment technique undertaken. All cells were cultured in a humidified environment at 37°C with 95% air and 5% carbon dioxide.

**Table 2:** Human cell lines used for this review and the corresponding culture media.

Cell line	Origin	Media	Enrichment Technique
U251 MG (Sigma Aldrich)	Glioblastoma	Eagle’s Minimum Essential Medium containing Earle’s Balanced Salt Solution (EMEM(EBSS)) (Sigma-Aldrich), supplemented with 2 mM L-glutamine (Sigma-Aldrich), 1% non-essential amino acids (NEAA) (Sigma-Aldrich), 1 mM sodium pyruvate (Sigma-Aldrich) and 10% fetal bovine serum (FBS) (Gibco, Paisley, UK).	OncoQuick®, Screen Cell®, pluriBead® and Cell Search®
PNT2 (Sigma-Aldrich)	Normal prostate	Roswell Park Memorial Institute (RPMI) 1,640 Medium (Sigma), 2 mM glutamine and 10% FBS	Cell Search®
VCaP (ATCC)	Prostate carcinoma	Dulbecco’s Modified Eagle Medium (DMEM) (ATCC 30–2002) plus 10% FBS;	Cell Search®
LNCaP (ATCC)	Prostate adenocarcinoma	Kaighn’s Modification of Ham’s F-12 Medium (ATCC 30–2004) plus 10% FBS.	Cell Search®
PC-3 (ATCC)	Prostate adenocarcinoma	Kaighn’s Modification of Ham’s F-12 Medium (ATCC 30–2004) plus 10% FBS.	Cell Search®
T24 (ATCC)	Urinary bladder transitional carcinoma	Modified McCoy’s 5a Medium (ATCC, 30–2007) plus 10% FBS.	Cell Search®
RT4 (ATCC)	Urinary bladder transitional carcinoma	Modified McCoy’s 5a Medium (ATCC, 30–2007) plus 10% FBS.	Cell Search®
TCCSUP (ATCC).	Bladder transitional-cell carcinoma	EMEM(EBSS), supplemented with NEAA plus 10% FBS.	Cell Search®
MCF10A (Sigma-Aldrich)	Normal breast	MEGM™ (Mammary Epithelial Cell Growth Medium) BulletKit™ (Lonza). The gentamycin-amphotericin B mix, provided with this kit was replaced with 100 ng/ml cholera toxin (Sigma).	Cell Search®
MCF7 (ATCC)	Breast adenocarcinoma	Modified EMEM (EBSS) (ATCC 30-2003) plus 10% FBS.	Cell Search®
Hs578T (ATCC)	Breast carcinoma	DMEM (ATCC 30–2002) plus 0.01 mg/mL human insulin (Gibco) plus 10% FBS.	Cell Search®
T47D (ATCC)	Breast carcinoma	Modified RPMI-1640 Medium (ATCC 30–2001) plus 10% FBS.	Cell Search®

All cells were cultured in a humidified environment at 37°C with 95% air and 5% carbon dioxide.

### 2.2 CTC enrichment techniques

#### 2.2.1 OncoQuick^®^ (Greiner Bio-One, Gloucestershire, UK)

**Figure 1:**
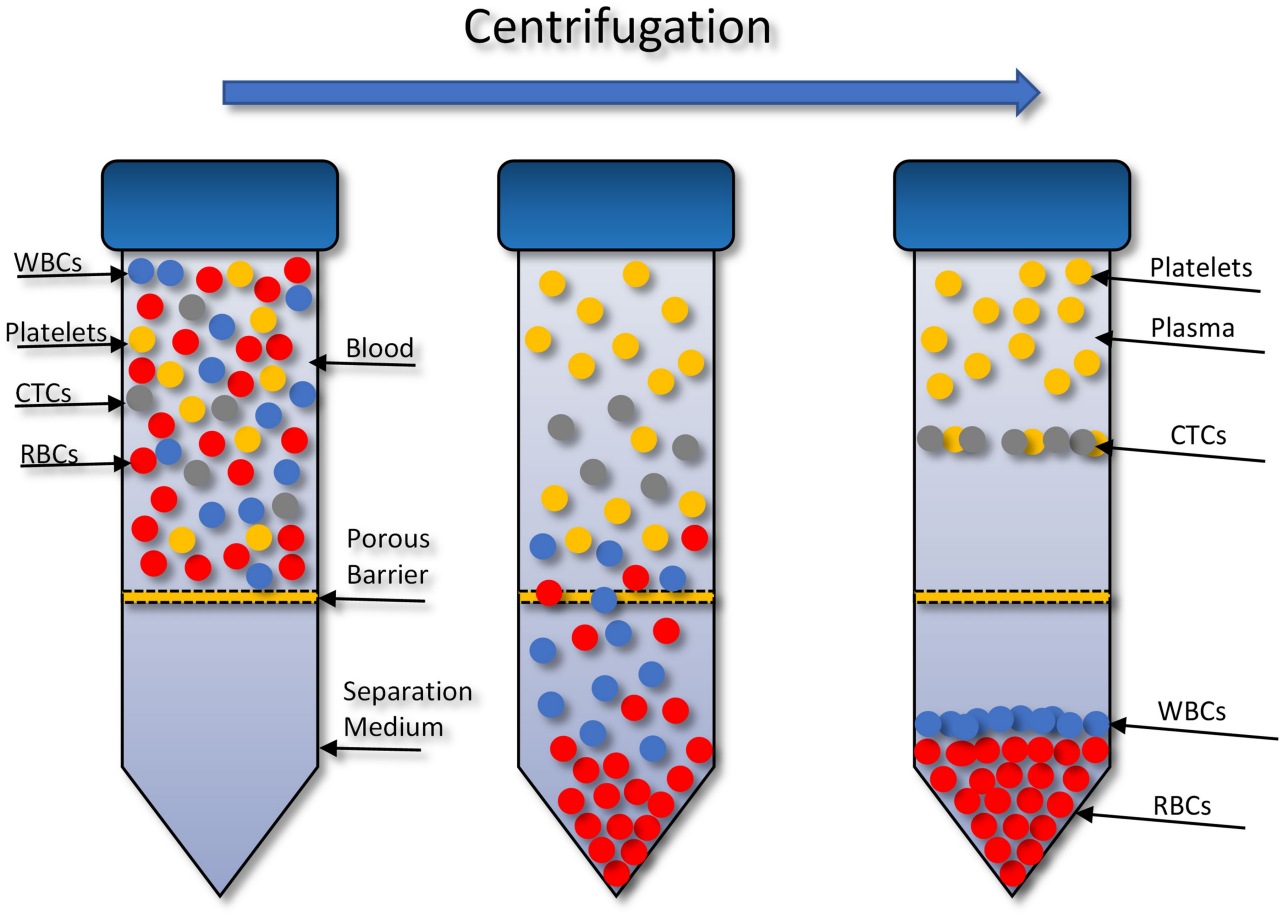
Circulating tumour cell (CTC) enrichment using the OncoQuick^®^ technique. This method uses density centrifugation combined with a porous barrier to isolate CTCs. RBCs, red blood cells; WBCs, white blood cells. Figure created with PowerPoint.

The OncoQuick® technique isolates CTCs through density gradient centrifugation (Figure 1). In 2014 (72), successfully isolated CTCs from high grade glioma patients using this method. To validate this method 15 ml of normal EDTA-anticoagulated whole blood was spiked with U251 MG cell densities: 1 × 10^4^ and 1.5 × 10^2^. Before starting the enrichment process the OncoQuick® tubes and normal whole blood samples were pre-cooled on ice for 10–15 min. The blood sample was then added carefully to the upper compartment of the OncoQuick® tube ensuring that the separation medium under the porous barrier was not disturbed. The OncoQuick® tube was then spun at 1,600 *g* for 20 min at 4°C, with a slow acceleration and no brake. Following centrifugation any captured tumour cells resided between the lower separation medium (blue) and the upper plasma (yellow). The liquid above the porous barrier was collected with a sterile serological pipette and transferred to a fresh sterile centrifuge tube. Walls of the OncoQuick® tube were carefully rinsed with 5 ml OncoQuick® wash buffer to collect any remaining tumour cells. This was then transferred to the centrifuge tube. Total volume in the new centrifuge tube was made up to 50 ml with wash buffer and the tube was inverted 5 times to mix the sample. Any cells present were pelleted by spinning the sample at 200 g for 10 min. The supernatant was removed leaving a pellet in 5 ml wash buffer. The pellet was re-suspended by carefully tapping the tube. This step was then repeated by adding another 45 ml of wash buffer. The supernatant was carefully aspirated without disturbing the cell pellet. The pellet was then re-suspended in growth media and transferred to a 24 well cell culture plate. Cells were then maintained in a humidified atmosphere at 37°C in 5% carbon dioxide.

#### 2.2.2 Screen Cell^®^ (Sarcelles, France)

**Figure 2:**
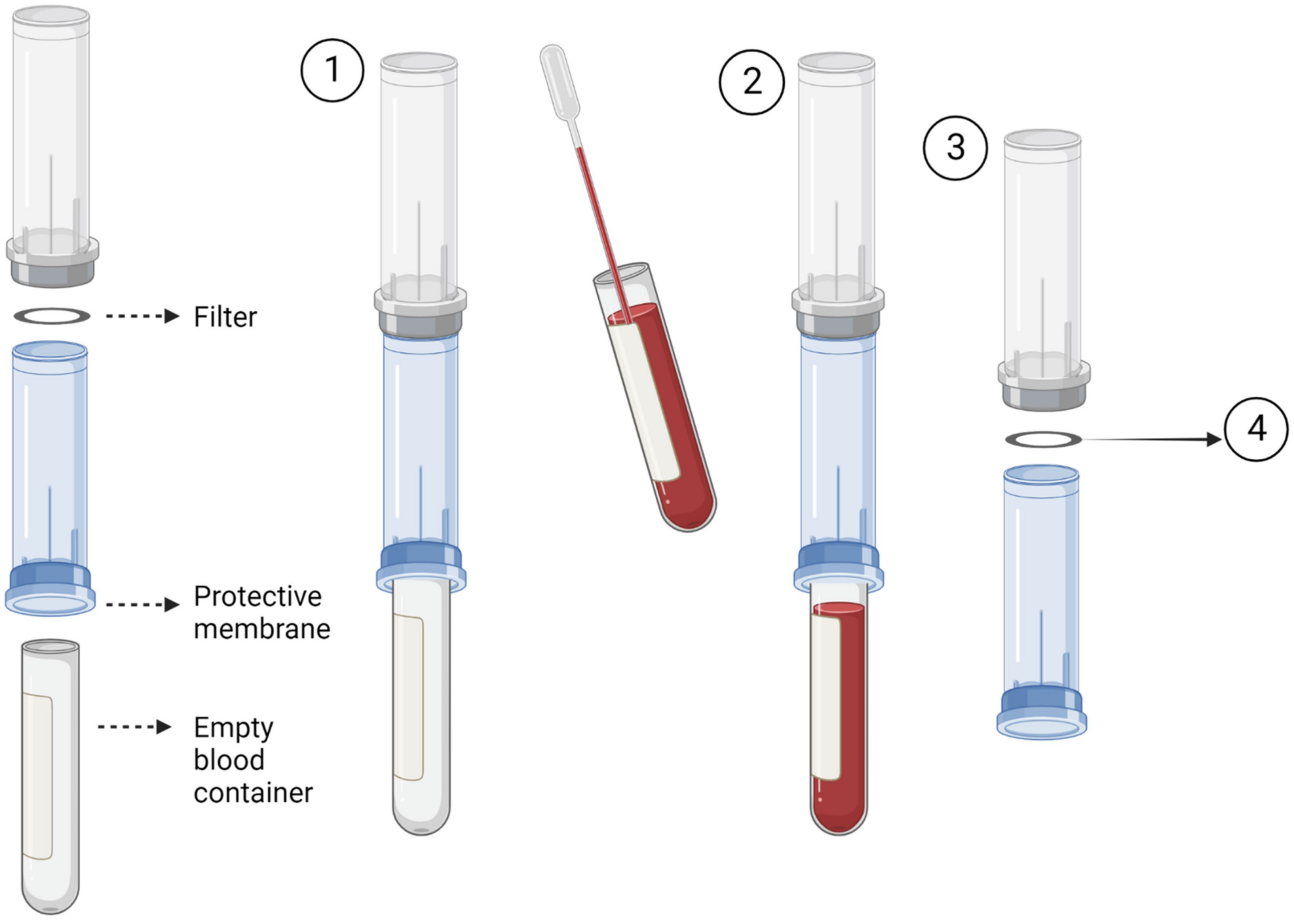
Circulating tumour cell (CTC) enrichment using Screen Cell^®^. This method captures the CTCs through size isolation on the surface of a membrane filter. A blood tube is inserted into the bottom of the device to create a vacuum (1). The pre-prepared blood sample is then added to the device. The blood then passes through the filter and is collected in the inserted blood tube (2). After the blood has fully passed through the filter, the device is separated (3). The filter is then removed and used for the desired test i.e., cell culture or molecular biology. Figure created with Biorender.

The Screen Cell® method captures CTCs through size isolation (Figure 2). As the blood sample travels through the filter, unlike the blood cells, the CTCs are too large to pass so are captured on the surface of the filter. To test this procedure, 3 ml of normal whole blood was spiked with U251 MG cell densities: 3 × 10^3^ and 2 × 10^1^. This was the most rapid method that was evaluated taking only 3 min to process the sample. Two different Screen Cell® kits were tested. Screen Cell®-Live Cell Detachment (LCD) kit is used to culture captured CTCs. Following filtration, the filter is released into a 24 well tissue culture plate and media is added. Cytological studies can be performed on the filter once the cells have adhered. Screen Cell®-Molecular Biology (MB) Kit is used for molecular biology examinations. Each pack includes a single DNAse and RNAse free filtration device, specialised buffer, and a collection tube. This unit enables DNA/RNA to be extracted directly from cells captured on the capsule’s filter or the cells can be cultured and subsequently analysed. Both kits followed the same procedure. The blood sample was transferred into a 15 ml sterile conical tube and 1 mL of Screen Cell® LC buffer was added to the sample. The tube was inverted 5 times and left to incubate for 3 min. For Screen Cell®-LCD samples only, 1.6 ml of growth media was added before the tube was inverted to homogenize. Before the blood samples were added to the device the protective membrane was removed, and a blood collection tube was placed underneath to create a vacuum. Following filtration, the device was carefully separated, and the filter was released into a 24 well plate. The plates were then maintained in a humidified atmosphere at 37°C in 5% carbon dioxide.

#### 2.2.3 Cell Search^®^ System (Janssen Diagnostics, South Raritan, USA)

**Figure 3:**
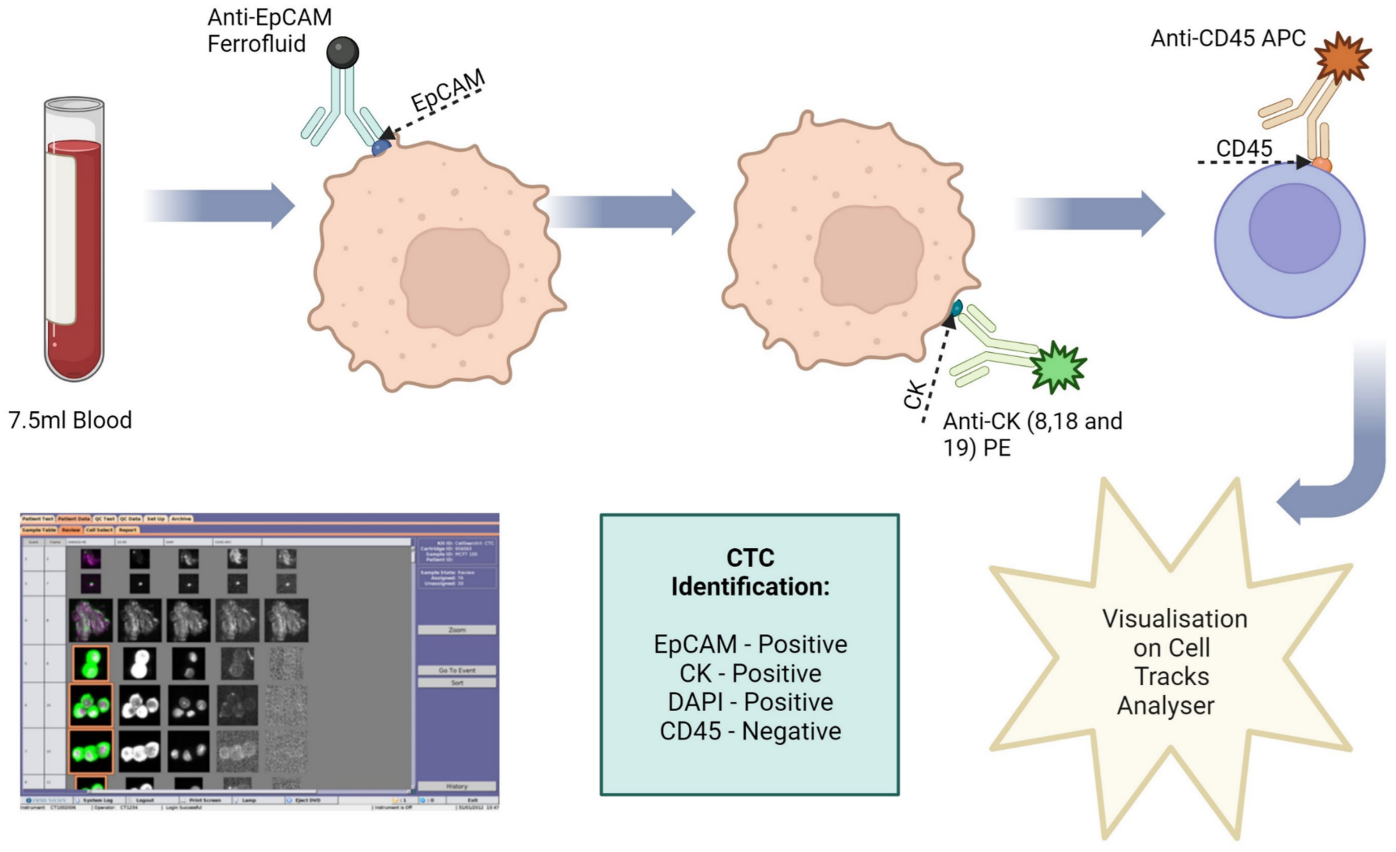
Circulating tumour cell (CTC) enrichment using the Cell Search^®^ System. This system uses anti-epithelial cell adhesion molecule (EpCAM) conjugated with magnetic beads to isolate CTCs. The cells are then stained with fluorescence-labelled monoclonal antibodies, which target cytokeratin (CK) 8, 18 and 19. CTCs are identified as epithelial cell adhesion molecule (EpCAM) positive, CK positive, 4′,6-diamidino-2-phenylindole (DAPI) positive and CD45 negative. Image made with Biorender.

The Cell Search® system uses an immuno-magnetic separation procedure to separate target cells (Figure 3). The cells are then stained with fluorescence-labelled monoclonal antibodies.

To validate this method 7.5 ml of normal whole blood was spiked with 1 × 10^4^ U251 MG cells. This is the minimum amount of blood required by the Cell Search® System. A CellSave Preservative Tube was used for blood collection, following which the tube was inverted 8 times to mix the sample with the anticoagulant and preservatives. The blood sample was then spiked with the U251 MG cells. Prior to processing, the spiked blood sample was transferred to a CellTracks® AutoPrep® System tube. The dilution buffer (6.5 ml) was added to the blood sample and the tube was inverted 5 times to mix. The samples were then centrifuged at 800 *g* for 10 min with no brake. During the run the system adds ferrofluid to the sample. Ferrofluid contains particles which have a magnetic core and are coated in monoclonal antibodies to bind to target cell antigens. The system adds a strong magnetic field to pull the labelled cells to the side and aspirates the blood. The magnetic field is then removed, and the cells are re-suspended in sample buffer. Another magnetic field is applied to separate the target cells from the wash buffer. Fluorescence-labelled antibodies are applied to bind to the target cell antigens and the cells are once more separated using a magnetic field. Finally, a cell fixative is applied, and the cells are transferred to a cartridge inside a specialised cell presentation fixture (MagNest®), through its strong magnetic field. The MagNest® is then loaded onto CellTracks Analyser II®, which identifies target cells through its fluorescent staining patterns.

#### 2.2.4 pluriBead^®^ (pluriSelect, Lepzig, Germany)

**Figure 4:**
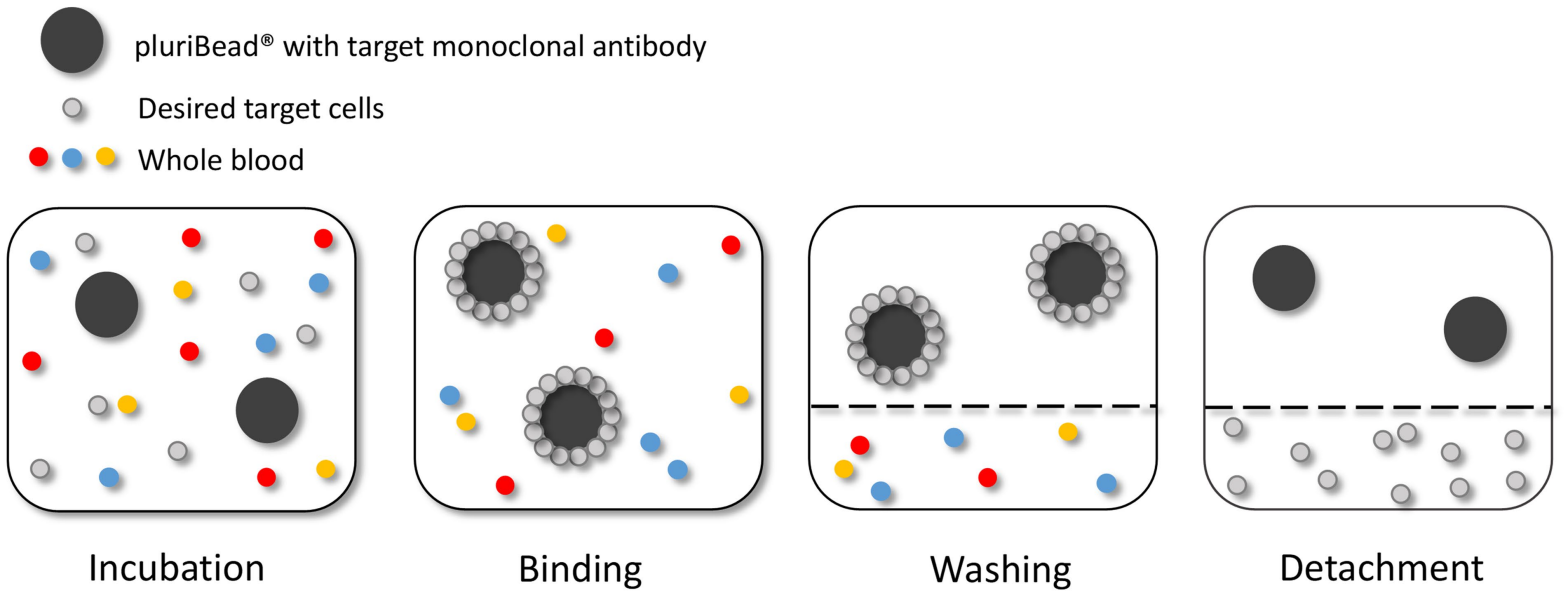
Circulating tumour cell (CTC) enrichment using the pluriBead^®^ technique. This method captures the CTCs using non-magnetic beads coupled with monoclonal antibodies specific to CTC surface antigens. Figure created with PowerPoint.

The pluriBead® method captures CTCs using non-magnetic beads coupled with monoclonal antibodies specific to CTC surface antigens (Figure 4). Six S-pluriBead® suspensions were developed specifically for this project. Antibodies selected were anti-EGFR, anti-mesenchymal-epithelial transition factor (c-MET) and anti-cadherin 11 (CDH11). Sullivan et al. (71), had previously isolated GBM CTCs by targeting these cell surface antigens. Six antibody clones were selected to maximise the chances of successfully capturing the cells: OB-Cadherin – clone N-12 (sc-30314, Santa Cruz Biotechnology, Heidelberg, Germany), OB-Cadherin – clone 16G5(ab151446, abcam, Cambridge UK), EGFR – clone 528(sc-120, Santa Cruz Biotechnology), EGFR – clone MGR1(ALX-804-572-C100, Enzo, Exeter, UK), EGFR – clone ICR10(ab231, abcam) and c-MET – clone EP1454Y (ab51067, abcam).

As recommended by the manufacturer the beads were initially tested with cells suspended in 3 mL growth media before spiking whole blood. The density of U251 MG cells used to test each bead was 3 × 10^3^. To validate each S-pluriBead® suspension, buffer A (150 μl) was added to 3 ml growth media, spiked with U251 MG cells. The S-pluriBead® suspension was vortexed and 120 μl were added to the sample, which was then left to mix for 30 min using a horizontal roller mixer. Following incubation, the S-pluriStrainer was placed on top of a sterile 50 ml centrifuge tube and equilibrated by adding 1 ml of wash buffer. The sample was then poured carefully onto the pluriStrainer to capture the beads with potential tumour cells attached. The beads were washed with 20 mL of wash buffer in 2 ml steps. The inner and outer surfaces of the strainer were washed to avoid target contamination. A connector was then attached to a fresh centrifuge tube and the luer-lock was closed. The strainer containing the beads was then attached to the connector, making sure the fit was tight. The beads were re-suspended in 1 ml of wash buffer and 10 μl of the suspension were placed in a 24 well plate to check whether any target cells were bound to the beads under a microscope. Activated buffer D was then added along the wall of the strainer and left to incubate for 10 min. Following incubation, 1 ml of wash buffer was added and the suspension was mixed 10 times with a pipette, making sure the mesh filter was not touched. The luer-lock was then opened, and the beads were washed with 10 ml of wash buffer. The connector and strainer were removed, and the cells were spun for 10 min at 300 g. The supernatant was carefully aspirated to leave 0.5 ml, making sure any potential pellet was not disturbed. Finally, 1 ml growth media was added to the potential pellet which was then re-suspended using a pipette and transferred to a 24 well plate. Cells were then maintained in a humidified atmosphere at 37°C in 5% carbon dioxide.

### 2.3 Cell characterisation

#### 2.3.1 Immunofluorescence

Before staining, the isolated U251 MG cells were grown on a μ-Chamber 8 well (Ibidi, Glasgow, UK) for 24 h at a density of 300 μl of 5 × 10^4^ cells/ml per well. Following this the cells were fixed in 3% paraformaldehyde (Alfa Aesar, Heysham, UK)/phosphate buffered saline (PBS) solution for 20 min and then permeabilised with 0.1% Triton-X100 (Thermo Fisher Scientific, Loughborough, UK)/PBS (150 μl/well) for 15 min, washing with PBS between each step. The cells were then blocked with 3% bovine serum albumin [BSA/PBS for 1 h and then incubated with the primary antibody, EGFR (EP38Y, abcam) diluted in 3% BSA at a ratio of 1:100 at room temperature for 1 h]. 3% BSA/PBS was used as a negative control. The cells were then washed in PBS before the secondary antibody, Alexa Flour 488 goat anti- mouse (Invitrogen, Thermo Fisher Scientific) diluted in 3% BSA/PBS at a ratio of 1:500, was applied for 1 h. The cells were then washed in PBS and one drop of mounting medium with 4′,6-diamidino-2-phenylindole (DAPI) (Vector Laboratories, Peterborough, UK) was applied to each chamber. The cells were then viewed using a fluorescent microscope at x40 and x100 magnification.

#### 2.3.2 Trypan blue exclusion

Total viable cell numbers were determined using the trypan blue exclusion assay. Following trypsinisation, resulting cell suspensions were mixed 1:1 with trypan blue dye and counted using a haemocytometer (Neubauer chamber). Trypan blue is excluded by viable cells, conversely cells that have undergone cell death have compromised cell membranes and therefore take up the trypan blue dye.

#### 2.3.3 Protein extraction and western immunoblotting

Total protein was extracted from cells using lysis buffer [10 mM tris hydrochloride (HCL) (Sigma), 50 mM sodium chloride (NaCl, Sigma), 5 mM EDTA (Sigma), 1% (v/v) triton X-100 (Sigma), 15 mM tetrasodium pyrophosphate (Sigma), 50 mM sodium fluoride (Sigma), 100 uM sodium orthovanadate (Sigma), phosphatase (Sigma, P5726), and protease (Sigma, P8340) inhibitors (10 ul/1 mL lysis buffer)].

Protein quantification was completed using a Pierce™ BCA (Bicinchoninic acid) Assay kit (ThermoFisher Scientific, 23227), iMark™ Microplate Reader (Bio-Rad, UK) and accompanying Microplate Manager® Software. 30ug of whole cell lysate were diluted 1:1 with laemmli x 2 sample buffer concentrate and 10% 2-mercaptoethanol (Sigma). Samples were then heated at 95°C for 5 min in an AccuBlock™ digital dry bath (Labnet International). After sodium dodecyl sulfate–polyacrylamide gel electrophoresis (SDS-PAGE), the separated proteins were transferred to a nitrocellulose membrane (Bio-rad, 1620094). Non-specific binding sites were blocked with 5% BSA in tris-buffered saline TWEEN®20 (TBS-T) for 60 min at room temperature. The membrane was then probed with EpCAM (ab32392, abcam) at a dilution of 1:2500 in 5% BSA overnight at 4°C before being washed in TBS-T and then incubated with anti-rabbit secondary (Sigma, A0545) at a dilution of 1:2000 in 5% BSA for 60 min at room temperature. Proteins were visualised by clarity enhanced-chemiluminescence (ECL) substrate (BioRad, 1,705,061) using BioRad Chemidoc XRS + system and analysed using Image Lab software (BioRad).

## 3 Results

### 3.1 OncoQuick^®^

**Figure 5:**
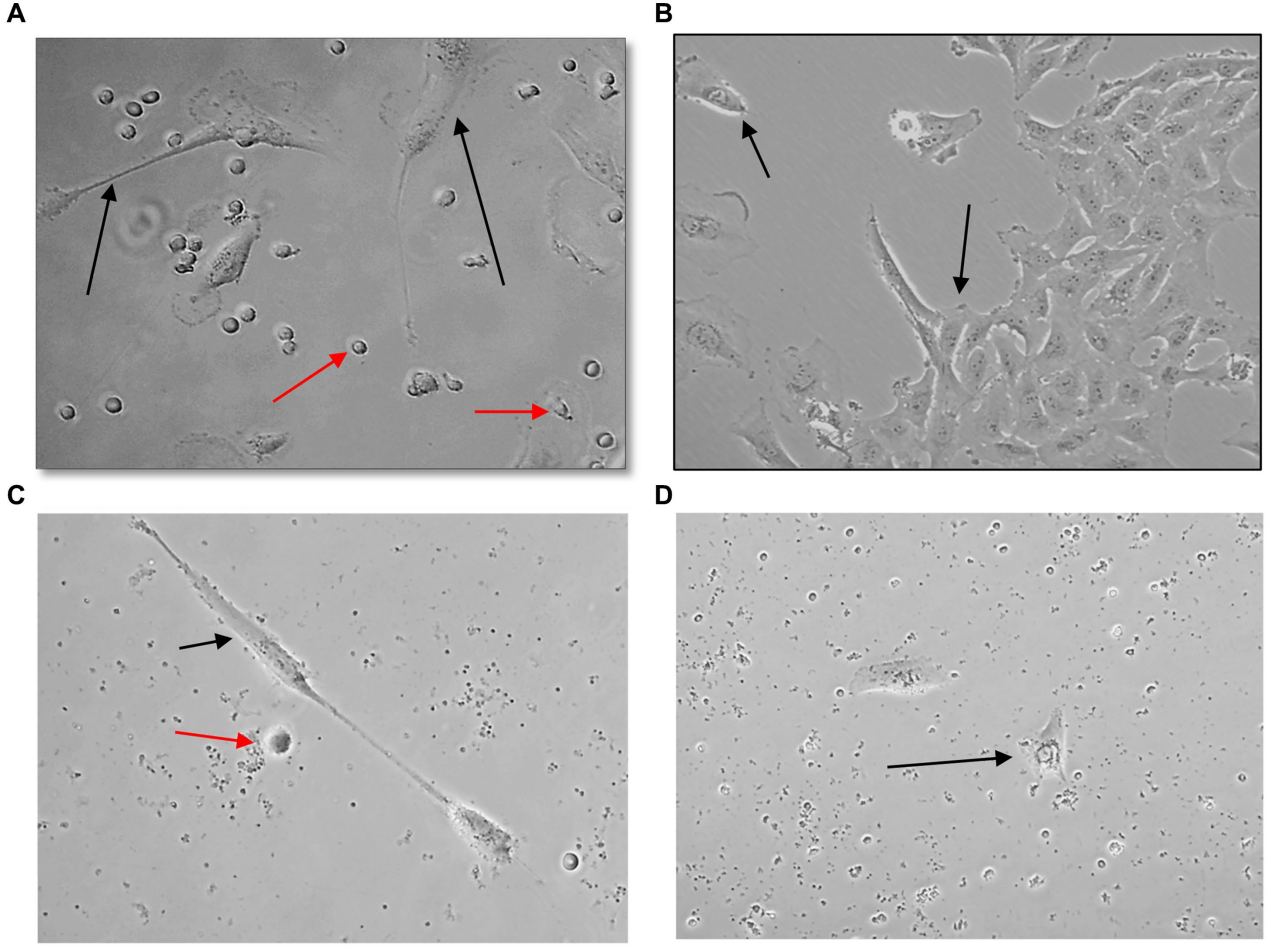
Captured U251 MG cells following OncoQuick^®^ processing of spiked whole blood. Images taken two **(A,C)** and seven **(B,D)** days after 1 × 10^4^ **(A,B)** and 1.5 × 10^2^ **(C,D)** U251 MG cells were spiked in 15 ml of normal whole blood and processed with the OncoQuick^®^ method. U251 MG cells (black arrows) were successfully seeded and cultured on 24 well plates. The red arrows highlight red blood cells which were also captured. x40 and x80 magnification.

**Figure 6:**
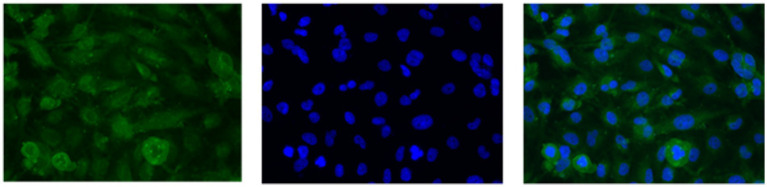
U251 MG cells captured with the OncoQuick^®^ technique, from spiked normal whole blood, were stained with immunofluorescence using the markers epidermal growth factor receptor EGFR (green, transmembrane protein) and 4′,6-diamidino-2-phenylindole (DAPI) (blue, nuclear marker). x40 magnification.

U251 MG cells were successfully isolated and cultured, when OncoQuick® processed 1 × 10^4^ and 1.5 × 10^2^ cells spiked in 15 ml of normal whole blood (Figure 5). The captured U251 MG cells were labelled using EGFR and DAPI immunofluorescence (Figure 6), suggesting that immunofluorescence could be used as an effective tool for CTC cell characterisation.

### 3.2 Screen Cell^®^

**Figure 7:**
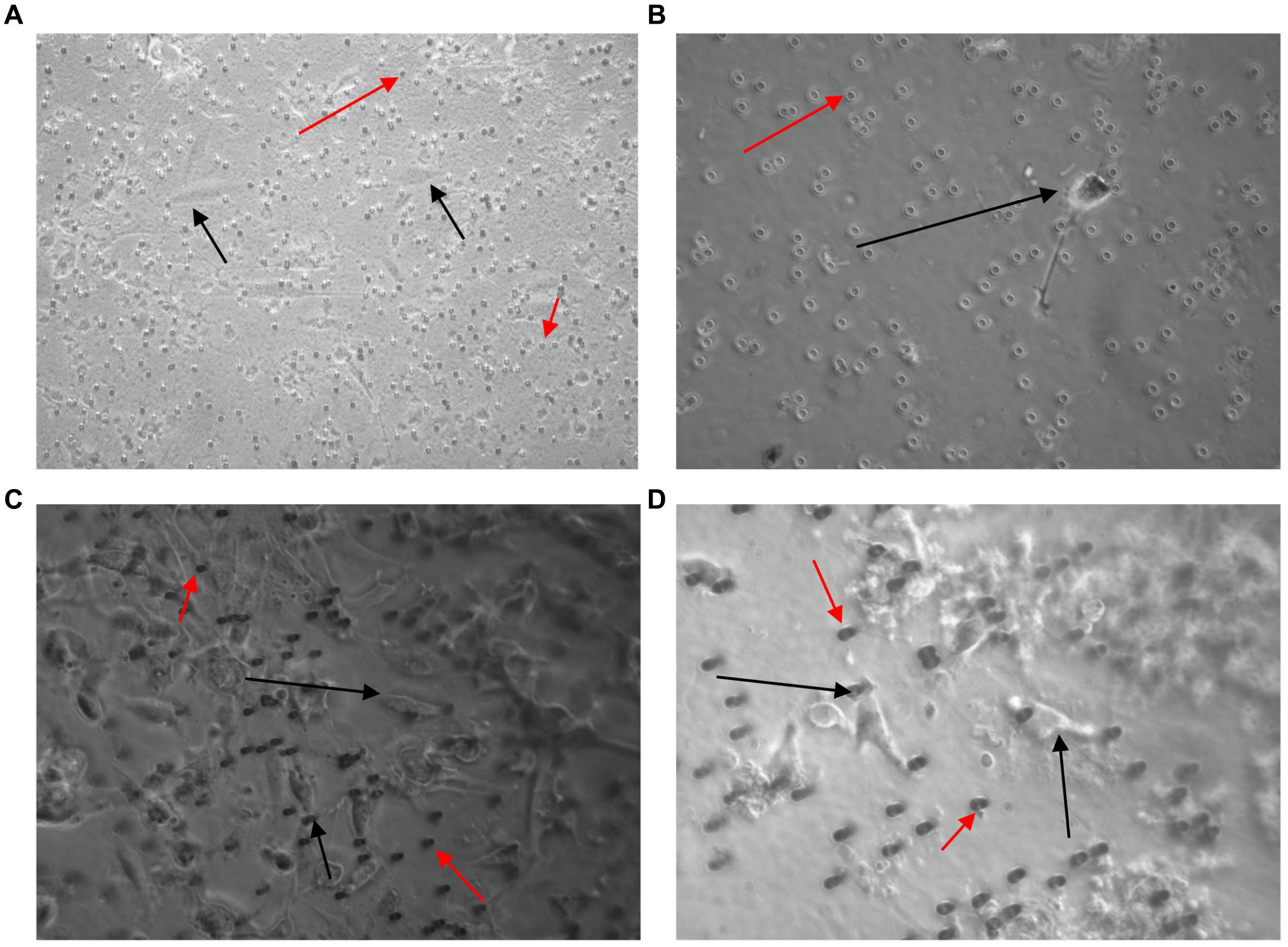
Images taken 7 days after 3 × 10^3^ **(A,C)** and 2 × 10^1^ **(B,D)** U251 MG cells were spiked in 3 ml of normal whole blood and processed using the Screen Cell^®^-LCD **(A,B)** and Screen Cell^®^-MB kit **(C,D)**. U251 MG cells (black arrow) were successfully seeded and cultured on the 24 well plate. The red arrow indicates blood cells, which were also captured. x40 and x100 magnification.

A high number of U251 MG cells were captured on the filter using the Screen Cell®- LCD kit and the cells were successfully cultured for 7 days (Figures 7A,B). To evaluate the sensitivity of Screen Cell®-CC the procedure was repeated with just 2 × 10^1^ cells in 3 ml of normal whole blood. In this case the procedure was successfully able to capture the U251 MG cells, which were then cultured (Figure 7). The Screen Cell®-MB kit was also used to successfully isolate, and culture 3 × 10^3^ and 3 × 10^2^ U251 MG cells spiked in 3 mL of normal whole blood (Figures 7C,D).

### 3.3 Cell Search^®^

**Figure 8:**
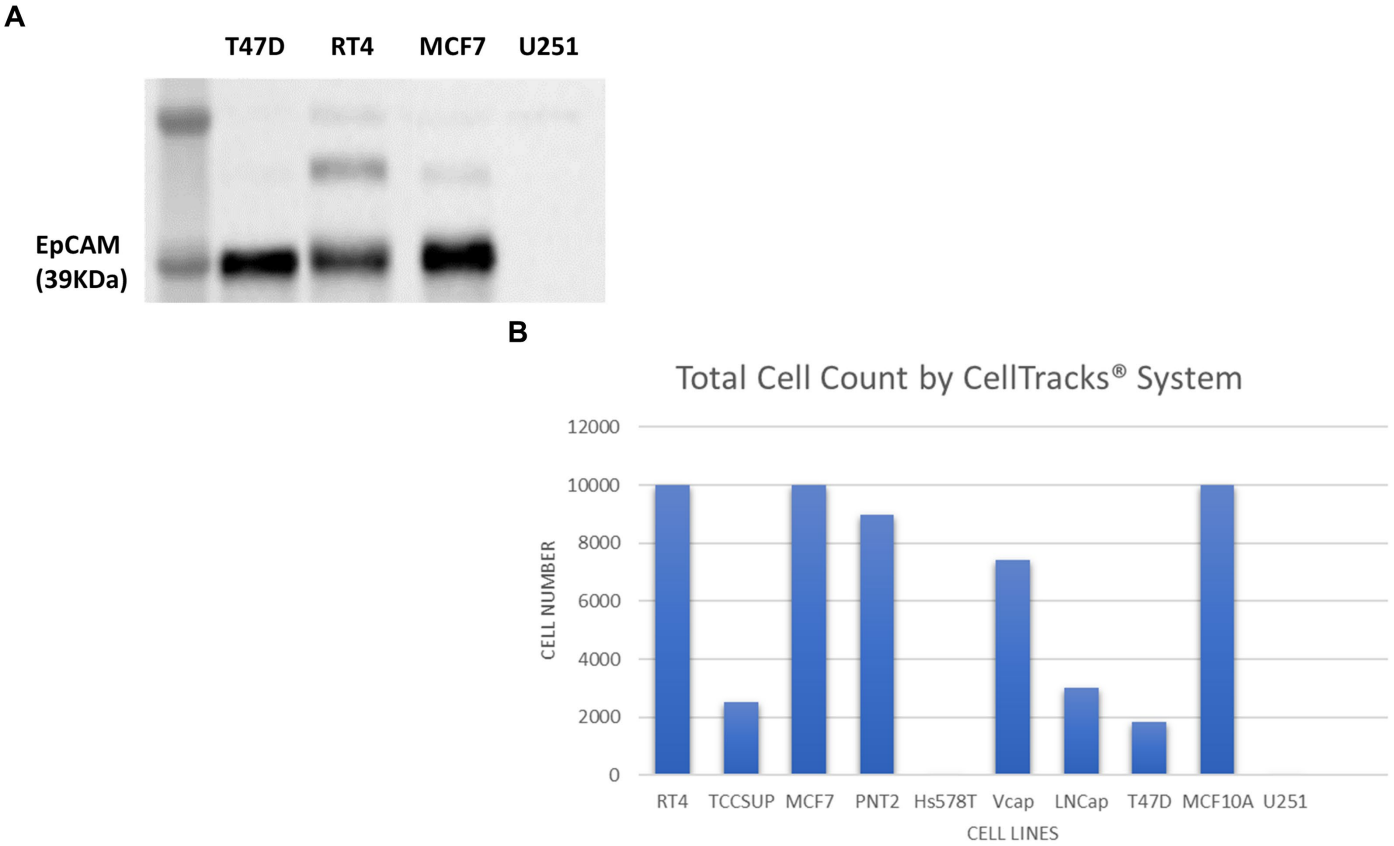
Immunoblot analysis of EpCAM protein expression in T47D (human breast carcinoma), RT4 (human bladder transitional-cell carcinoma), T24 (human urinary bladder transitional carcinoma), MCF7 (human breast adenocarcinoma) and U251 MG (human glioblastoma) cell lines **(A)**. The number of cells identified by the Cell Search^®^ System when 1 × 10^4^ cells, from RT4, MCF7, T47D, TCCSUP (human bladder transitional-cell carcinoma), PNT2 (normal human prostate), Hs578T (human breast carcinoma, ATCC), VCaP (human prostate carcinoma, ATCC), LNCaP (human prostate adenocarcinoma) and MCF10A (normal human breast) were spiked into 7.5 ml normal blood samples **(B)**.

The Cell Search® System uses EpCAM to detect and enumerate CTCs. Although EpCAM is absent in the healthy brain tissue, a study by Chen et al., identified that not only was there an overexpression of EpCAM in gliomas, but it also correlated significantly with malignancy (103). To determine whether EpCAM was present in U251 MG cells SDS-PAGE electrophoresis and western blotting was conducted (Figure 8A).

In contrast to cell lysates from T47D (human breast carcinoma), RT4 (human bladder transitional-cell carcinoma) and MCF7 (human breast adenocarcinoma) cells, analysed alongside the U251 MG cells, no EpCAM was detected in the U251 MG cell lysate or in the T24 (human urinary bladder transitional carcinoma) cell line.

To confirm these findings using the Cell Search® System, U251 MG cells were run alongside the following cells lines RT4, MCF7, T47D, TCCSUP (human bladder transitional-cell carcinoma), PNT2 (normal human prostate), Hs578T (human breast carcinoma), VCaP (human prostate carcinoma), LNCaP (human prostate adenocarcinoma), MCF10A (normal human breast). Each sample contained 7.5 ml of normal whole blood spiked with 1 × 10^4^ cells. Figure 8B demonstrates the number of EpCAM positive cells detected for each cell line. As anticipated no U251 MG cells were detected by this system compared to the MCF7 cells, for example, which had demonstrated EpCAM positivity in the western blot.

### 3.4 pluriBead^®^

**Figure 9:**
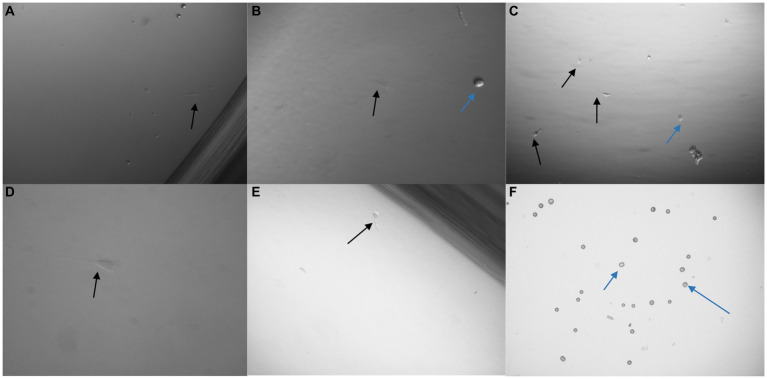
Images taken 48 h after 3 × 10^3^ U251 MG cells were processed with a non-magnetic bead suspension each coupled with a different monoclonal antibody: EGFR clone ICR10 **(A)**, OB-Cadherin clone N-12 **(B)**, c-Met clone EP1454Y **(C)**, EGFR clone MGR1 **(D)**, OB-Cadherin clone 16G5 **(E)** and EGFR clone 528 **(F)**. On completion of the pluriBead^®^ technique the cell suspensions were seeded on a 24 well plate in cell media and cultured at 37°C and 5% carbon dioxide. An average of only 2 U251 MG cells (black arrow), (*n* = 3), were detected with each antibody clone, except EGFR (clone 528) despite the media initially being spiked with 3 × 10^3^ U251 MG cells. The blue arrows indicate beads which were also noted. x40 magnification.

The pluriBead® method was first analysed with 3 × 10^3^ U251 MG cells suspended in growth media. Each set of beads, with a separate clone of antibody adhered to it, was tested. Prior to detachment, 10 μl of each solution was taken and pipetted onto a 24 well plate so that it could be checked under a microscope to see if any U251 MG cells had adhered to the beads. No U251 MG cells could be detected at this stage with any of the antibody clones. Following processing the cell pellet was re-suspended in growth media and seeded onto a 24 well plate. The plate was then examined 48 h later to see if any U251 MG cells had been successfully captured (Figure 9). An average of only 2U251 MG cells (*n* = 3) were detected with each antibody clone, except EGFR (clone 528) despite the media initially being spiked with 3 × 10^3^ U251 MG cells.

**Figure 10:**
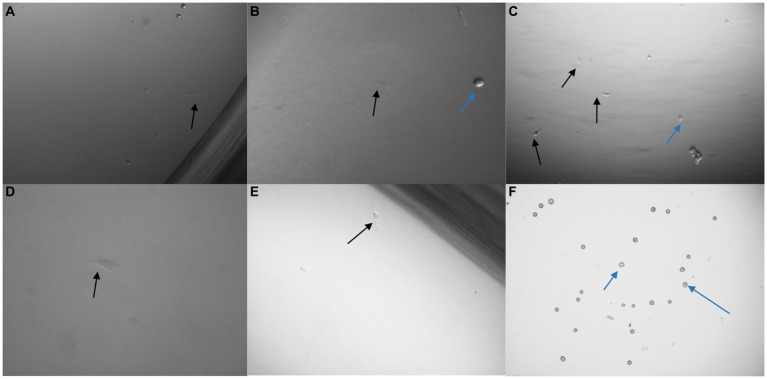
3 × 10^3^ PC3 cells were processed with a non-magnetic bead suspension coupled with six separate monoclonal antibodies EGFR clone 528 **(A,B)**, OB-Cadherin clone N-12 **(C,E)**, EGFR clone ICR10 **(D)**, OB-Cadherin clone 16G5, EGFR clone MGR1 and c-Met clone EP1454Y **(F)**. **(A–C)** Taken prior to detachment phase of the pluriBead^®^ technique. **(D–F)** Taken 72 h after pluriBead^®^ cell suspensions were cultured in cell media at 37°C and 5% carbon dioxide. x40 and x100 magnification. Black arrows depict PC3 cells and the blue arrows indicate beads observed. PC3 cell counts were undertaken using trypan blue dye exclusion assay 72 h after pluriBead^®^ technique.

To further determine the effectiveness of the pluriBead® method the technique was repeated with an alternative cell line PC3 (prostate adenocarcinoma, Sigma-Aldrich). On completion of this technique a much higher yield of PC3 cells had been captured for each antibody. A trypan blue exclusion assay was undertaken to determine the number of viable cells successfully isolated by each monoclonal antibody (Figure 10).

The pluriBead® used were designed specifically for this study. The markers, EGFR, c-Met and CDH11, which have previously been used successfully to capture GBM CTCs, were selected to isolate the cells (71). When viewing the beads, prior to the detachment stage, it appeared that no U251 MG cells had been successfully captured despite processing 3 × 10^3^ cells. A very small number of cells (≈2 cells) were noted growing 48 h later, however this could be due to contamination rather than successful capture by the beads.

## 4 Discussion

The Cell Search® System was unable to detect U251 MG cells spiked in normal whole blood due to the lack of EpCAM on the surface of these cells. Modifications could be made to the system to include additional markers, but at present only EpCAM positive cells are detected by this system.

Other studies have shown the failure of the Cell Search® System to detect rare CTCs and also detect CTCs in patients with a widely metastatic disease, despite those patients have high numbers of CTCs identified through alternative methods (94, 104, 105).

Tumour progression is associated with a loss of epithelial features and a transition towards a mesenchymal phenotype, a process known as EMT. Tumour cells are known to undergo EMT as a means of entering circulation (106). A loss in epithelial markers, such as EpCAM, would prevent the CTCs from being detected by the Cell Search® System. Konigsberg et al. (107), determined, in metastaic breast cancer patients, that the density gradient centrifugation method OncoQuick® appeared advantageous for CTC isolation compared to MACS HEA MicroBeads® (MACS), which also relied on EpCAM immunomagmetic enrichment technology. This technology also requires the costly purchase of the Cell Search® System, which could prevent many clinical settings from using this system.

The U251 MG cells were successfully enriched using the OncoQuick® method, which correlates with the findings from (72), who successfully isolated CTCs from high grade glioma patients using this method. Although the method was successful, many steps were required to perform the analysis, which is time consuming. This is an important factor to consider when reviewing this technique for clinical application. Numerous steps would also increase the chances of losing ‘rare’ CTCs, particularly when transferring the solution between centrifuge tubes. The manufacturer recommends that the plasma fraction is discarded when platelet contamination is seen following centrifugation. Removing the plasma fraction could result in loss of CTCs due to unwanted contamination of this fraction. Alternatively, CTCs could form non-specific aggregates, which could cause them to move to the bottom gradient, again leading to false negative results. CTCs have been shown to bind with platelets, fibroblasts, and leukocytes to evade blood stream hazards (108, 109). If CTCs are present in these clusters, they could also move to the bottom gradient and subsequently be missed through the OncoQuick® method.

GBMs have a high degree of intratumoral heterogeneity (110). CTC profiles can change during tumour cell dissemination (111–113). Before entering the blood CTCs undergo varying degrees of EMT, which leads to variability in cell markers (114). Although the selected markers for the pluriBead® technique are associated with tumorigenesis and cell migration in GBMs, c-Met acting as an independent predictor for GBMs, they are heterogenous (115–117). The successful capture of PC3 cells compared to U251 MG isolation, suggests that the selected markers were not present on the surface of the U251 MG cells. This also highlights the potential difficulty of attempting to capture CTCs in a clinical setting using the pluriBead® technique.

To isolate GBM CTCs using purely biological properties rather than their physical properties increases the chances of CTCs being missed. Multiple clones of the same primary antibody were selected for the pluriBead® technique. One clone typically binds to just one target molecule presenting a single epitiope. The epitopes present could vary greatly in each CTC, even from the same parent tumour, which would minimise the successful chances of capturing CTCs with pluriBead®. To increase the possibility of capturing CTCs, the pluriBead® technique could be repeated overall several rounds with beads conjugated to different antibodies. This however also increases the chances of losing CTCs, particularly when there could just be one CTC present in the blood sample. This would also increase the time required for the completion of this technique.

Both Screen Cell®- LCD and Screen Cell®-MB kits were able to isolate U251 MG cells, which were then successfully cultured. The Screen Cell® method was easy to use and rapid, taking only 3 min to perform the process. The technique is also sensitive, capturing cells from a blood sample, which had been spiked with just 2 × 10^1^ U251 MG cells. Another benefit of the method is the single enrichment step, consisting of blood passing directly through a filter, which may reduce the chance for CTCs to be lost. Both kits offer the advantage of supporting further analysis of markers. The Screen Cell®-MB kit has a particular advantage that the cells can be analysed directly for DNA/RNA, or they can be cultured first and then analysed for DNA/RNA. This quick and cheap method could be undertaken at the patient’s bedside with no requirement for pre-processing. Fast enrichment also minimises disruption to the CTCs, which preserves the cell phenotype.

Cell counts used for the validation of the enrichment techniques used in this study were as recommended by the manufacturers. A clear limitation of this study is that the cell counts are much higher than those expected when if clinical samples were tested with the selected method. Nonetheless this has provided us with a good opportunity to assess the limitations of each method, even with a higher cell count. Bang-Christensen et al. (78), reported enriching between 0.5 and 42 CTCs in 3 ml blood. Therefore 3 mL of normal whole blood spiked with 2 × 10^1^ U251 MG cells was used to test the sensitivity of the ScreenCell® technique. The ScreenCell® method was successfully able to isolate the U251 MG cells at this concentration. Another limitation of this study is that commercial cultures rather than patient derived cultures have been used. Additionally, the techniques were not validated on patient plasma samples.

In comparison to the other enrichment methods compared in this study, Screen Cell® appears most favourable to use in a healthcare setting as it is simple, cheap and quick to use (Table 3). Only one step is required before the CTCs are captured on the membrane filter, through size isolation, thus, maximising the chances of CTC capture. This method could be easily introduced into a busy clinical setting, where reliable and quick results are required. This system offers an option for simple cytomorpholical diagnosis after routine staining of CTCs. It also supports a number of other potential characterisation techniques and could enable captured CTCs to be successfully cultured.

**Table 3:** Advantages and disadvantages of the OncoQuick^®^, ScreenCell^®^, Cell Search^®^ and pluriBead^®^ enrichment techniques.

**Enrichment Technique**	**Rationale**	**Advantages**	**Disadvantages**
**OncoQuick®**	Enumeration by combined density-based centrifugation and filtration. Integration of a porous barrier above the separation media captures CTCs and enables smaller white and red blood cells to pass through.	Label-free technique, which captures modified and viable cells. Cost effective. Successfully captured U251 MG cells. Captured cells were successfully cultured and characterized using immunofluorescence. Cultured cells could be used for xenografting in immunocompromised mice. Able to process 15 mL of whole blood using this method, which may increase chances of capturing CTCs.	Multi-step process, cells can be lost and time consuming (difficult in a busy clinical setting). Contamination with blood cells noted. CTCs could be lost in supernatant.
**ScreenCell®**	Enrichment using a membrane microfilter	Simple, convenient, cost effective. No mechanical damage to cells. Successfully captured U251 MG cells. Cells maintain viability so can be cultured subsequent to processing. Captures cells based on size separation using a single step process, therefore less chance for CTCs to be missed. Technique suitable for a busy clinical setting. Method enables CTC cells to be cultured after enrichment and captured cells can be used for molecular typing, genetic profiling and xenografting.	Contamination with blood cells noted. Manufacturer recommends using a 3 mL blood sample, which may decrease chances of capturing CTCS.
**Cell Search®**	Immunomagnetic enrichment with ferrofluid nanoparticles that target EpCAM. Characterisation with cytokeratin (8, 18+, and 19+) monoclonal antibodies. CD45 antibody used to differentiate white blood cells from CTCs.	Automatic technique. Simple, convenient and easy to operate. No pre-treatment required. Highly sensitive and reproducible. First and only clinically validated, FDA approved, blood test for enumerating CTCs.	Costly, need to purchase instrument as well as Cell Search® components. Did not capture U251 MG cells, − only enriches CTCs with cell surface EpCAM. CTCs are widely heterogenous. Relying on cell surface markers for enrichment could lead to false negative results.
**pluriBead®**	Enumeration by antibody coated beads, which target CTC surface antigens. The pluriBeads® are bound to CTCs are then sieved to isolate cells from whole blood. CTCs can then be removed from beads for characterisation.	Method is sensitive and quick. High purity of CTCs. CTCs can be cultured following enrichment. A variety of characterisation techniques can be under taken on the isolated CTCs including molecular typing and genetic profiling.	Relies on cell surface markers to isolate CTCs. CTCs are heterogenous so a higher chance of missing the CTCs. Multi-step process so cells could be easily lost. To date no universal CTC antigens have been identified. Only a very small number (≈ 2) of U251 MG cells captured through this method. This could be potentially due to contamination rather than the beads successfully capturing the cells. Manufacturer recommends using a 3 mL blood sample, which may decrease chances of capturing CTCS.

CTCs, circulating tumour cells; EpCAM, epithelial cell adhesion molecule.

By contrast, isolation methods which rely on single CTC biomarkers such as pluriBead® and the Cell Search® System could lead to higher false negative results, due to CTC heterogeneity. EGFR for example, which was used to characterise isolated U251 MG cells, demonstrates heterogeneous expression in GBMs (115). A multi-step process such as the OncoQuick technique could also lead to CTCs being missed, due to them being lost during one of the processing stages.

The potential benefit of using GBM CTCs diagnostically in the healthcare setting is threefold: it could enable earlier diagnosis, disease monitoring and potential reassurance of the worried well. Out of the four commercially available CTC enrichment methods – OncoQuick®, Screen Cell®, pluriBead® and Cell Search®- we found that the Screen Cell® method offered the most potential for translational application in the clinical setting. Alongside being simple, cheap and quick, this CTC enrichment method was not limited to isolating CTCs through one characteristic. It also supports a wide range of downstream analysis options. Further validation of the ScreenCell® technique is now required, which will be completed on GBM patient blood samples in a clinically relevant setting.

## Data availability statement

The original contributions presented in the study are included in the article/supplementary material, further inquiries can be directed to the corresponding author.

## Ethics statement

The studies involving humans were approved by The Brain Tumour Bank South West and Brain UK Ethics 15/006. The studies were conducted in accordance with the local legislation and institutional requirements. The participants provided their written informed consent to participate in this study.

## Author contributions

HB: Conceptualization, Data curation, Formal analysis, Investigation, Methodology, Project administration, Validation, Visualization, Writing – original draft, Writing – review & editing, Funding acquisition. CP: Supervision, Writing – original draft, Writing – review & editing. KK: Supervision, Writing – original draft, Writing – review & editing, Conceptualization, Funding acquisition.
